# Consumer Profile and Drivers Influencing Consumer Behavior towards *Fondillón*, a European Protected Naturally Sweet Red Wine

**DOI:** 10.3390/foods10112651

**Published:** 2021-11-01

**Authors:** Hanán Issa-Issa, Luis Noguera-Artiaga, María Mora, Ángel A. Carbonell-Barrachina, David López-Lluch

**Affiliations:** 1 Research Group “Food Quality and Safety”, Centro de Investigación e Innovación Agroalimentaria y Agroambiental (CIAGRO-UMH), Miguel Hernández University, Carretera de Beniel, km 3.2, 03312 Orihuela, Spain; hissa@umh.es (H.I.-I.); luis.noguera.umh@gmail.com (L.N.-A.); 2 BCC Innovation, Technology Center in Gastronomy, Basque Culinary Center, 20009 Donostia-San Sebastián, Spain; mmora@bculinary.com; 3 Basque Culinary Center, Faculty of Gastronomy Sciences, Mondragon Unibertsitatea, 20009 Donostia-San Sebastián, Spain; 4 Departamento de Economía Agroambiental, Ingeniería Cartográfica y Expresión Gráfica en la Ingeniería, Centro de Investigación e Innovación Agroalimentaria y Agroambiental (CIAGRO-UMH), UMH, Ctra. Beniel, km 3.2, 03312 Orihuela, Alicante, Spain; david.lopez@umh.es

**Keywords:** Alicante wine, consumer acceptance, descriptive sensory analysis, *Monastrell*, oxidized wine

## Abstract

*Fondillón* is a naturally sweet red wine, protected within the Alicante Denomination of Origin (Alicante, Spain) and recognized by the European Union in its E-Bacchus database. The aims of this study were (*i*) to evaluate the degree of consumer acceptance and satisfaction towards *Fondillón*, (*ii*) to establish key drivers controlling consumer satisfaction, and (*iii*) to establish a general profile of the typical *Fondillón* consumer. The experiment consisted of three complementary studies: (*i*) a descriptive sensory analysis of five *Fondillón* samples representing all samples being marketed, (*ii*) an affective test using wine consumers (*n* = 100), and (*iii*) an online questionnaire to identify the main characteristic of a *Fondillón* consumer (*n* = 294). The main consumption drivers were good balance, intense floral and fruity notes, and long aftertaste. The current typical *Fondillón* consumer is a 42–52 year-old man, with a higher education level, with a 25,000–50,000 euros/year income, and who drinks it mainly at home. The online study showed that 50% of respondents do not consume *Fondillón* because they do not know it, because it is very expensive, or because it is not so easy to find. Therefore, producers should improve their communication campaigns and distribution networks as key parts of their marketing strategies regarding *Fondillón*.

## 1. Introduction

Wine is an alcoholic beverage that is consumed worldwide. Wine quality is determined by several factors such as cultivar, environmental conditions, quality of raw materials at harvest, and winemaking process, especially storage conditions and hygiene. All these factors transform each wine into a unique product with aromatic and gustatory complexity and typicity [1].

A special group of wines are those with residual sugar; they include a large range of styles. Some inexpensive wines contain low to moderate levels of residual sugar, making them appealing to a larger consumer segment. There are different ways of producing wines with residual sugar (*i*) by concentrating the sugar in the grape must (overripening of the grape); (*ii*) stopping the fermentation before dryness (adding alcohol or by freezing), or (*iii*) blending in a sweetening component (e.g., adding must) to the wine. The method selection will be determined by the vineyard climate, the local wine legislation, the wine style and quality, and the final market price that the winemaker wants for their products. In any case, the global objective is to produce a wine with balanced residual sugar and acidity, and with a flavor profile appropriate for the final sugar content [2].

Concentrating the sugar in the grape must is the method used to produce many sweet and luscious wines. The concentration of sugars in the grape must be sufficient to stop the fermentation before reaching dryness; yeasts struggle in very sugary environments, especially when alcohol is also present, and naturally stop fermentation even at relatively low levels of alcohol. These wines are known as *naturally sweet wines*. This process not only concentrates sugar, but also other grape components such as organic acids and flavor precursors. The increase in sour and flavor components helps in keeping the balance of these wines by avoiding that sweetness becomes too dominant; this is the key reason why many of these wines have an outstanding organoleptic quality. In general, these wines can have more complexity than those simply made by stopping the fermentation by chilling or adding a sweetening component [3]. However, these grapes are difficult to process, because (*i*) the volume of juice obtained from the grapes is low, and (*ii*) the very sugary pulp is also often hard to extract during pressing. These factors must be added to the normal production cost, making them only appropriate to be sold for premium and super premium wines with a “high selling price”. In fact, there is a growing interest in high-quality naturally sweet wines [4], but this is not supported by a deep research on consumer preferences and drivers controlling sweet wine consumption [5], especially if this research is compared with the number of papers on dry wine consumption. This lack of research is hampering a full development of suitable marketing strategies by wineries producing sweet wines, including those producing *Fondillón*.

*Fondillón* is a naturally sweet wine made only in the province of Alicante and having a set of specific characteristics that makes it unique. Its main features are: (*i*) it comes from the overripening of *Monastrell* (also known as Mourvèdre) grapes, with very high amounts of sugars, aroma precursors, and color components; (*ii*) its alcohol content is entirely produced by fermentation of the grape sugars, that is, no fortification takes place at any stage; (*iii*) the minimum alcohol allowed is 16° abv (alcohol by volume); and (*iv*) it must be aged in oak barrels for, at least, 10 years [6]. *Fondillón* production was almost lost at the end of the 19th century due to, among other reasons, a high demand of cheap wine from Europe because of *Phylloxera*. Production was recovered around 1950; however, there has not been an appropriate approach to understand the special chemistry behind this wine and to promote it among non-highly-specialized wine consumers [7].

The perception of distinction is not an objective process but a social one taking place in a field of cultural production [8]. Consumers look for and are willing to pay high prices for wine if its symbolic position allows for their social distinction [9]. In this way, the initial working hypothesis of this study was that consumers do not know the peculiarities and singular characteristics of *Fondillón* mainly because (*i*) consumers with high purchasing power were not interested on this specific wine from a non-trendy wine PDO (Alicante) due to a lack of scientific information on its peculiarities, and (*ii*) consequently, winemakers lost their interest in producing and selling this wine, especially due to the long inversion in time needed for its production. However, this situation is changing and the few manuscripts characterizing *Fondillón* [10] and comparing it to other high-quality sweet wines, such as *Tokaj* and *Sauternes*, are getting winemakers and consumer attention and making this wine more and more popular again. As a result, in the last two-three years the *Fondillón* selling price in wine shops has increased by about 30–40%, showing that consumers are starting to be aware of its uniqueness and willing to buy more and more *Fondillón* bottles and this higher demand has made winemakers concentrate on this wine and has even made them quick to renew the *Fondillón* packaging to communicate to consumers its exclusivity. In this way, 11,050 bottles of *Fondillón* were labeled under the Alicante PDO in the season 2019–2020 as compared to only 5720 in the season 2014–2015, showing an increase of ~93% (data kindly provided by the Regulatory Council of the Alicante PDO). In 2020, a *Fondillón* (*Brotons Gran Fondillón Reserva* 1964) was included among the best Spanish wines and this prize (Best Wine 2020, Spanish Ministry of Agriculture, Fisheries and Food) has also positioned this Alicante wine in the focus of the specialized media. 

Consequently, the aims of this study were: (*i*) to evaluate the degree of consumer acceptance and satisfaction towards *Fondillón*, (*ii*) to establish key drivers controlling consumer satisfaction, and (*iii*) to establish a general profile of the typical *Fondillón* consumer, including the main reasons why *Fondillón* is not as popular as other naturally sweet wines, such as *Sherry*, *Porto*, or *Madeira*. This information will be essential for Alicante winemakers to understand consumer behavior towards *Fondillón* (buying and satisfaction drivers) and adjust their marketing/selling strategies to help *Fondillón* recover its symbolic position of social distinction and make consumers order this wine in modern restaurants, particularly in those specialized in Mediterranean cuisine.

## 2. Materials and Methods

### 2.1. Wine Samples

In 2019, only 9 wineries in Alicante produced *Fondillón* under the PDO Alicante. To reduce the number of samples to be evaluated, a preliminary grouping of the commercial *Fondillón* samples was conducted using a napping test with 20 trained panelists. They classified wines into 5 clusters (the main characteristics/sensory descriptors of each cluster are described below); one sample from each of these clusters was selected and fully evaluated in the current study. Samples (3 bottles of 750 mL for each sample) were kindly provided by the Regulatory Council of the Alicante PDO. The samples analyzed were:

(i) **F1**, *Gran Reserva* 1987 (sweet, Muscat, raisins, and amber color).
(ii) **F2**, *Solera* 1948 (sweet, bitter, woody, paperboard, and toffee).
(iii) **F3**, *Reserva Especial* (bitter, sour, alcohol, and mahogany color).
(iv) **F4**, *Gran Reserva* 1964 (sweet, bitter, roasted, toffee, and long aftertaste).
(v) **F5**, *Solera* 1996 (sweet, roasted, caramel, vanilla, and amber color).

### 2.2. Descriptive Sensory Analysis

For the sensory evaluation of *Fondillón* samples, the tasting panel of the Regulatory Council of the Alicante PDO was used. This panel consisted of 12 panelists (6 women and 6 men), aged between 30 and 60 years old, with more than 400 h of experience in describing Alicante wines. These panelists were (*i*) selected (3 sessions of 1.5 h), according to results in discrimination, ranking, and recognition tests, (*ii*) trained (12 sessions of 1.5 h sessions, for 4 months), and (*iii*) validated (2 sessions of 1.5 h). The panel is included in the control tools of the Regulatory Board to monitor the quality of their wines and is accredited by ENAC (National Entity of Accreditation), following the norm ISO/IEC 17065:2012 [11]. Panelists were paid for their involvement in the current study and any other evaluation they performed. A maximum of 10 wines can be tasted in a single session, including 1 repeatability (same sample evaluated twice in the same session), 1 reproducibility (same sample evaluated in two different sessions), and 1 defect sample.

The vocabulary used to describe the *Fondillón* samples was that of the official lexicon of the PDO Alicante and the attributions, definitions, and reference materials are described in Issa-Issa et al. [7]; these attributes were selected by the panel during the lexicon development. The attribute “Mediterranean forest” replaced “vegetable” because during the last 3 years of experience of the panel, it was demonstrated that the term vegetable was too general and did not properly describe the notes found in *Fondillón* samples; a new reference material was prepared and consisted of rosemary dried at 40 °C for 24 h and its intensity was 8.0. The main attributes used for the evaluation of *Fondillón* samples were classified into 4 complex properties: appearance, odor, basic tastes, and flavor. In the appearance, only color was evaluated. Regarding odor and flavor (olfactory and gustative phases), the analyzed attributes were alcohol, fruity/nutty, floral, Mediterranean forest, spicy, animal, toasted/caramel, and chemical, together with the basic tastes: sweetness, sourness, and bitterness. Finally, astringency and aftertaste or persistence (time that the characteristic *Fondillón* wine flavor remains in the mouth after swallowing the sample) were also studied. 

The 5 samples under analysis were run in triplicate in 3 sessions of ~2.0 h. The deviation of quality control samples must be below 20% for all sensory descriptors. A maximum of 10 samples can be evaluated in a single session of 2.0 h, including 3 quality control samples (reproducibility, repeatability, and sample with a defect). Samples were randomly served coded with 3-digit numbers, together with the appropriate questionnaire, one at a time, and with waiting 10 min between samples. Initially, ~35 mL of each sample were served in black cups at 16–18 °C for the evaluation of the flavor and later ~20 mL were served in transparent cups in normalized booths at a controlled temperature of 22 ± 2 °C. To avoid carry over effects, mineral water and breadsticks were provided as palate cleansers. 

To quantify the intensity of each of the attributes studied, a scale from 0 to 10 was used, with increments of 0.5 units; where, 0 indicates extremely low or no intensity, and 10 indicates extremely high intensity.

### 2.3. Affective Sensory Analysis

This study was carried out to establish the degree of overall consumer satisfaction, together with the satisfaction degree for key and easy-to-understand-by-consumers sensory attributes: color, alcohol (odor and flavor, *o* and *f*), toasted/caramel/coffee (*o*, *f*), chemical (*o*, *f*), sweetness, sourness, and aftertaste. A preference test was also conducted. The affective study was carried out with 100 Spanish consumers, recruited at the Orihuela campus of the UMH, and who drank sweet or oxidized wine (*Porto*, *Madeira*, *Sherry*, *Fondillón*, etc.) at least twice a month. The consumer profile was as follows: 44% women and 56% men, of which 20% were between 18 and 24 years old, 40% between 25–35, 20% between 36–44, and 20% above 45 years old.

All samples were coded using 3-digit numbers and served to each of the consumers one at a time in a random order. Wine service conditions (cups, wine temperature, and palate cleansers) were the same as those previously described for the descriptive study. A questionnaire was developed, where a liking for each of the studied attributes was evaluated, using a 9-point hedonic scale (9 = like it extremely, 5 = neither like it nor dislike it, and 1 = dislike it extremely). The intensity of the main *Fondillón* attributes were evaluated using a 9-point JAR (Just About Right) scale (1 = much too weak, 5 = just about right, and 9 = much too strong). Additionally, to identify the main sensory attributes driving overall liking, a Check-All-That-Apply (CATA) ballot was used; a list of 18 notes, including 9 odor and 9 flavor notes (fruity, vanilla, coffee, floral, caramel, nutty, Mediterranean forest, raisins, and toffee) was built for the intuitive description of the *Fondillón* samples by consumers; these 20 attributes were obtained from the preliminary napping test carried out to group wine samples and, thus, reduce the number of samples to be used in descriptive and affective studies. Finally, consumers were asked about (*i*) their preference (after tasting these 5 *Fondillón* samples, which sample did you like the most or prefer? This question was located at the end of the affective questionnaire and after the consumers tasted the 5 *Fondillón* samples); and, (*ii*) purchase intention (are you willing to buy this wine? This question was located at the end of the affective test for each one of the samples).

### 2.4. Online Survey

An online study (with 294 consumers, consisting of 62% men) was designed and conducted to determine the consumers niche of *Fondillón* and to understand the main reasons for the consumption or no-consumption of this wine. Participants were from two neighboring regions (Valencia and Murcia, comprising 4 provinces: Castellón, Valencia, Alicante, and Murcia) and should drink (*i*) Alicante PDO wine at least twice a week, or (*ii*) sweet or oxidized wine (*Porto*, *Madeira*, *Sherry*, *Fondillón*, etc.) at least twice a month. In this way, consumers were expected to have a basic knowledge or information on *Fondillón* due to their geographical proximity to its producing area; in further studies, consumers from all Spanish regions will be included, but initially only consumers from these two regions were targeted. The questionnaire had questions related to the consumption of *Fondillón*, and demographic questions to classify them according to gender, age, income, and education (Table S1).

### 2.5. Statistical Analysis

Results were subjected to a one-way analysis of variance (ANOVA) and then, a LSD multiple-range test was performed on the attributes analyzed with the trained panel and on liking. Differences were considered statistically significant at *p* < 0.05. Partial least square regression (PLS) was performed using sensory descriptors and consumer overall liking. JAR data were analyzed by a penalty analysis, which was carried out to obtain information on those weak points where the sensory profile of each of the samples can be improved. The normal distribution of the hedonic data was checked using the Jarque-Bera test for each sample. CATA results were analyzed using a Correspondence Analyses (CA) plot, which was created through a contingency table with 20 and 300 columns and rows, respectively. To perform all these statistical analyses, the software XLSTAT Premium 2016 (Addinsoft, Nueva York, NY, USA) and Statgraphics Plus (version 3.1, Statistical Graphics Corp., Rockville, MA, USA) were used.

## 3. Results and Discussion

### 3.1. Descriptive Sensory Analysis

A trained panel was used to develop the descriptive sensory profiles of the *Fondillón* samples under analysis; these profiles will be later used to determine which of their attributes are key drivers of consumer acceptance. In this way, Issa-Issa et al. [7], in a preliminary affective study on *Fondillón*, concluded that Spanish consumers appreciated samples with a high intensity of fruity notes, high alcohol content, and some bitter and Mediterranean forest notes.

Twenty-two sensory attributes were analyzed, with 16 of them showing significant differences (Table 1). It is important to highlight that sample F4 was the one that had the highest intensity (>5.0) of most of the attributes evaluated, including sweetness, alcohol (*o*, *f*) and fruity/nutty, and aftertaste, while sample F1 was characterized by a high intensity of color and toasted/caramel notes. 

Vitispirane (having as sensory descriptors eucalyptus and camphor) and TDN (1,1,6-trimethyl-1,2-dihydronaphthalene, which has sensory descriptors of petroleum or smoke) were identified as potential markers of the *Fondillón* age [7]. These two compounds can be linked with the descriptors (*i*) Mediterranean forest, which includes balsamic (vitispirane), and (*ii*) chemical (TDN) notes. Perhaps concerning the aging intensity, related attributes (Mediterranean forest and chemical) could be one of the factors affecting consumer acceptance and satisfaction. In this way, sample F4 showed the highest intensity of the attribute “chemical” (odor and flavor: *o*, *f*), which includes the petroleum note (TDN); on the other hand, there were not significant differences for the attribute “Mediterranean forest” (vitispirane).

### 3.2. Affective Sensory Analysis

Table 2 shows the overall satisfaction degree of Spanish consumers, and their liking regarding key sensory attributes of these wines, including color, alcohol (*o*, *f*), toasted (*o*, *f*), sweetness, sourness, and aftertaste.

The main parameter to be studied was the overall liking; that is, the “global” opinion of the consumer when tasting the wine samples F4 (6.9) and F1 (6.7) were the most liked ones, with scores close to 7.0 (like moderately); this value can be taken as a relatively high acceptance because consumers tend not to use the extreme values of the scale. Comparing the overall satisfaction degree for the 100 consumers participating in this study, it was shown that only 5% of the consumers who liked the F4 sample also liked the F1 sample. 

Moving to the liking of each of the 10 main sensory attributes selected for this study, samples F1 and F4 were those showing the highest satisfaction scores. For instance, the acceptance of sample F1 was based on high liking scores (>6.2), for all studied attributes except by chemical (*o*). This sample (F1) was defined by consumers in their comments as a balanced wine, easy to drink, fresh, and clean. This description could be linked to the high score observed for the satisfaction degree on sourness; besides, the high score (6.6) on the aftertaste liking indicated that despite the long ageing—above 10 years—no off-flavor notes were perceived by consumers. The high overall liking scores of the F4 sample were based on high scores for color, sweetness, toasted (*f*), and aftertaste; in this case, aftertaste can play a key role because it is very long, pleasant, and it is linked with the complexity of this special Alicante wine.

**Figure 1:**
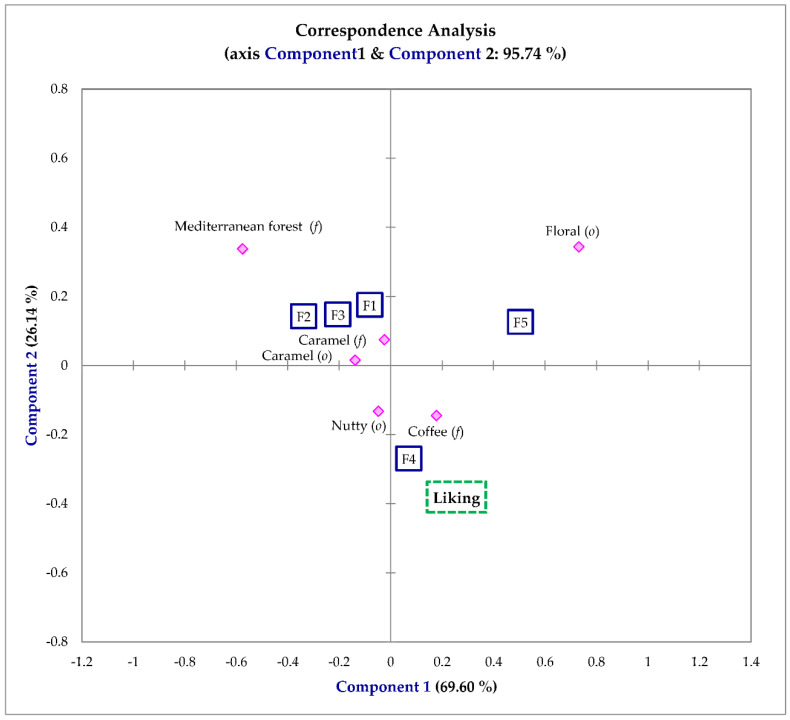
Differences among the samples according to the CATA descriptors used to study the Fondillón samples under study (Correspondence Analysis). Legend: ☐ wine samples; ◇ sensory attributes. --- consumers´ overall liking.

Figure 1 shows the first two components of the correspondence analyses (CA) plot, which explained 95.74% of the CATA data variability; three groups can be recognized based on the position of the samples in the CA. The first group included sample F4, which was linked with nutty (*o*) and coffee (*f*) notes. A second group consisted of samples F1, F2, and F3, which had more relevant descriptors such as caramel (*o*, *f*) and Mediterranean forest (*f*). Finally, sample F5 was characterized by its floral (*o*) note. 

Results from the preference test showed that the two most preferred samples were F4 and F1 (30 and 28%, respectively); while on the other hand, only 10, 13, and 18% of consumers preferred samples F2, F3, and F5, respectively. Finally, and regarding the purchase intention, 100 and 83% of the consumers were willing to buy samples F4 and F1, respectively, while ~60% were willing to buy the other three samples (60, 60, and 53% for F3, F5, and F2, respectively).

### 3.3. Partial Least Square Regression (PLS) and Correspondence Analysis

**Figure 2:**
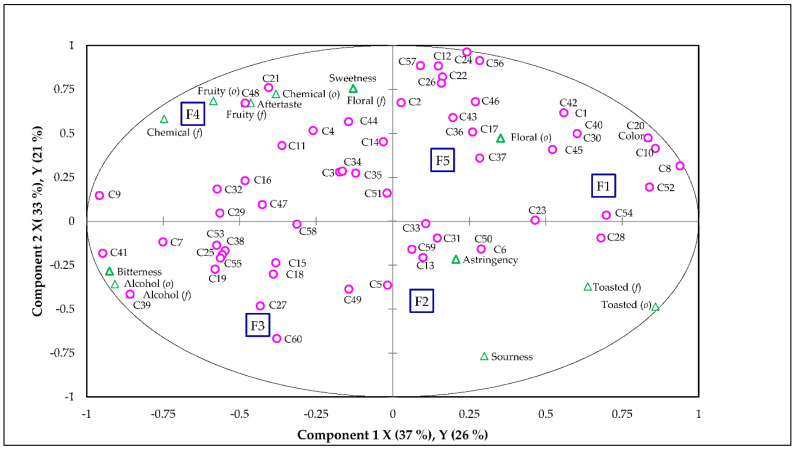
Partial Least Squares (PLS) regression of descriptive sensory attributes (X−axis) and consumers overall liking (Y−axis) of *Fondillón* samples under analysis. Legend: C consumers; ☐ Sample; △ sensory attributes, ○ consumer’s overall liking.

A PLS analysis was performed to analyze whether descriptive data was able to properly explain consumer overall liking (Figure 2). For this analysis, only those descriptive attributes being statistically affected (*p* < 0.05) by the type of *Fondillón* were included. It is important to mention that the low number of samples used in the study, five, limits the ability to assess relationships, but the number of samples was limited by the complexity and long aftertaste of the *Fondillón* samples analyzed. In this way, 70% of the variation of the descriptive data (X−axis) explained 47% of the variation of the consumer overall liking (Y−axis). Figure 2 shows that ~50% of consumers liked sweet *Fondillón* samples having floral (*o*) notes (F1 and F5). On the other hand, ~40% of consumers liked *Fondillón* samples being sweet, with fruity (*o*, *f*) notes, having characteristic chemical notes (*o*, *f*), and having a long aftertaste (F4). 

In general, the PLS analysis demonstrated that *Fondillón* consumers do not like samples with high intensity of bitterness, sourness, astringency, and/or alcohol or toasted/caramel notes (*o*, *f*) (F2 and F3). This experimental statement agreed with results from penalty analysis, indicating that the key drivers controlling consumer satisfaction towards *Fondillón* [12] were long aftertaste, high sweetness, and low alcohol and sourness (Figure S1). These consumer opinions/demands (limiting excessive sourness and alcohol notes) can be linked to some of the *Fondillón* samples slightly starting to be too oxidized, with sugars starting to be converted into organic acids, such as acetic acid [13], providing an slightly higher sourness, decreasing their characteristic sweetness and complexity, and starting to lose their balance.

Correspondence Analysis (CA) was used to evaluate the CATA results and showed the key role of the following descriptors: coffee, floral, caramel, nutty, and Mediterranean forest/rosemary (Table 3). Coffee and caramel notes (CATA) can be linked to the descriptive attribute “toasted/caramel” considering their definitions; in a similar way, the nutty note can be linked to the descriptive attribute “fruity” (reference material benzaldehyde, which has a sensory descriptor of bitter almond) [7]. This linkage among affective CATA notes and descriptive sensory attributes were initially established to understand which descriptive attributes were more easily perceived by consumers. Future studies with a higher number of consumers will be conducted to confirm these relationships, including volatile compounds generating the sensory attributes/descriptors. 

### 3.4. Consumers Profile

**Figure 3:**
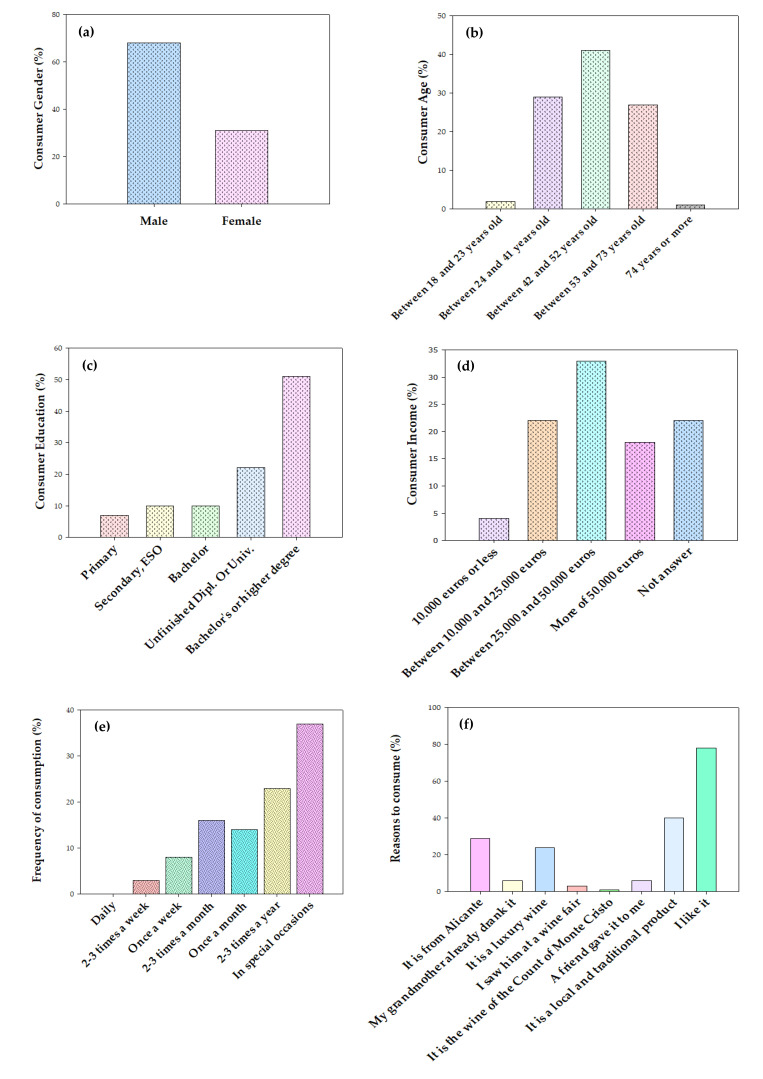
*Fondillón* consumer profile as affected by (**a**) gender, (**b**) age, (**c**) educational level, and (**d**) annual income, together with key consumption habits: (**e**) consumption frequency and (**f**) main reasons to buy and consume this wine.

In this online study, results showed that 50% of the respondents were *Fondillón* consumers, although all participants drank Alicante wine, at least twice a week, and oxidized wine, at least twice a month. This first result is important because it highlights the fact that *Fondillón* is not so popular, even for people consuming other types of Alicante wines and living in regions close to its production zone (Alicante PDO). The profile of the *Fondillón* consumers (not from all respondents but only those consuming *Fondillón* regularly) was as follows: 68% men (Figure 3A), of which 29% were between 24 and 41 years old, 41% between 42 and 52, and 27% between 53 and 73 years old (Figure 3B). Regarding the education and socioeconomic levels of consumers, 51% and 33% of the *Fondillón* consumers have bachelor or higher degree of education and an income between €25,000 and €50,000 (Figure 3C,D). The results also showed that only 3% of the participants drank *Fondillón* 2–3 times a week, 8% once a week, 16% 2–3 times a month, 14% 1 time a month, 23% 2–3 times a year, and 37% consumed it only on special occasions (Figure 3E). The reasons why they consume this type of wine were that (*i*) they like its characteristic flavor (78%), (*ii*) it is a local and traditional product (40%), (*iii*) it is an exclusive wine from the province of Alicante (29%), and (*iv*) it is a luxury wine (24%) (Figure 3F). The main sensory attributes driving consumers to drink this wine were sweetness, toasted/caramel aroma, raisin aroma, nutty aroma, long aftertaste, and its balance. In the specific case of consumption, 77% of the people surveyed answered that they only consumed *Fondillón* at home, while only 16% consumed it in restaurants. A potential explanation for this experimental finding can be the high price of *Fondillón* at restaurants or the fact that most of the restaurants do not have this type of wine on their wine list.

The reasons why wine consumers do not consume *Fondillón* are: (*i*) it is very expensive (9%), (*ii*) it is difficult to find (14%), (*iii*) it is not in the stores where they usually buy alcoholic beverages, including wine (27%), or (iv) simply because they do not know it (18%).

## 4. Conclusions

The sensory profile of the most liked *Fondillón* samples was: (*i*) good balance between alcohol, sweetness, and bitterness, (*ii*) intense floral and/or fruity notes, and (*iii*) a long and pleasant aftertaste. When wine consumers try *Fondillón*, they like it and are willing to keep drinking it, as happened with samples *F4* and *F1*. The main problem is that a wide percentage of consumers still do not know or have never tried *Fondillón*. The typical *Fondillón* consumer is a man between 42 and 52 years old, with bachelor or higher degree, and earning between 25,000 and 50,000 euros/year. They drink *Fondillón* mainly during special occasions at home and the two main reasons why they drink it are: (*i*) it is a traditional Alicante wine and (*ii*) because of its characteristic and distinctive flavor. The reasons for not ordering *Fondillón* at restaurants are: (*i*) its high price, and (*ii*) it is not generally included on the restaurant wine list. Producers should develop their marketing strategies regarding *Fondillón,* focusing on communication and distribution and keeping in mind that the two major constraints limiting its consumption are consumer value perception (consumers are not fully aware of the real value and high quality of *Fondillón* and then consider it is too expensive) and the lack of availability in both on and off trade. Further studies at the Alicante PDO can be conducted to study the profile of tourists visiting these wineries and their opinion on *Fondillón* after their first tasting, as has been done with similar wines, such as *Sherry* [14] and Porto [15] wines. Additionally, in future studies, the number of consumers participating in the affective test will be increased and consumers from all Spanish regions will be included, and then, the approach used to link descriptive and affective data will be preference mapping. 

## Figures and Tables

**Table 1:** Descriptive sensory profile of the *Fondillón* samples under analysis.

Attribute	ANOVA ^†^	F1	F2	F3	F4	F5
		LSD Multiple Range Test ^‡^				
**Appearance**						
Color	***	6.0 a	4.0 ab	3.0 c	4.0 ab	5.0 b
**Odor**						
Alcohol	***	5.3 b	6.1 ab	6.6 a	6.5 a	5.7 ab
Fruity	**	4.8 b	5.2 ab	5.3 ab	5.4 ab	5.7 a
Floral	*	2.0 a	1.6 ab	0.9 b	1.0 b	2.0 a
Mediterranean forest	NS	1.9	0.9	0.8	0.8	1.7
Spicy	NS	3.3	3.6	3.4	3.7	3.0
Animal	NS	1.3	1.4	1.4	0.9	1.3
Toasted	**	5.8 a	4.9 ab	5.1 ab	4.2 b	4.2 b
Chemical	**	2.2 c	2.7 b	2.0 c	3.2 a	2.7 b
**Basic taste**						
Sweetness	***	5.4 a	4.0 b	3.2 c	5.3 a	3.7 bc
Sourness	***	3.5 b	3.4 b	4.3 ab	3.4 b	3.8 ab
Bitterness	***	1.6 bc	2.1 b	3.3 a	1.1 c	1.5 bc
**Flavor**						
Alcohol	**	6.1 b	6.7 a	6.7 a	6.7 a	6.1 b
Fruity	**	5.7 a	4.9 ab	5.5 a	5.0 ab	4.5 b
Floral	*	1.4 b	0.9 b	2.1 a	1.1 b	2.3 a
Mediterranean forest	NS	1.3	0.9	1.2	0.9	1.5
Spicy	NS	3.6	4.0	3.5	3.4	3.8
Animal	NS	0.9	1.1	1.6	0.8	1.2
Toasted	***	7.2 a	6.5 b	5.7 c	5.8 c	4.8 d
Chemical	**	2.5 b	3.0 ab	2.7 b	3.7 a	3.0 ab
Astringency	***	0.8 b	1.6 a	1.2 b	0.8 b	1.0 b
Aftertaste	**	5.7 b	5.5 b	6.0 ab	6.7 a	6.0 ab

^†^ NS = not significant at *p* < 0.05; *, **, ***, significant at *p* < 0.05, 0.01, and 0.001, respectively. ^‡^ Values (mean of 12 trained panelists) followed by the same letter, within the same row, were not significantly different (*p* < 0.05), according to LSD least significant difference test.

**Table 2:** Liking scores of Spanish consumers regarding the *Fondillón* samples under analysis.

Samples	Overall	Color	Alcohol	Toasted	Chemical	Sweetness	Sourness	Alcohol	Toasted	Chemical	Aftertaste
			(*o*) ^¥^					(*f*) ^¥^			
	ANOVA Test ^†^										
	***	***	***	***	**	***	***	***	**	**	***
	**LSD Multiple Range Test ^‡^**										
F1	6.7 a	6.8 ab	6.8 a	6.8 a	5.4 ab	6.2 a	6.6 a	6.5 a	6.8 a	6.0 ab	6.6 ab
F2	5.8 b	6.5 ab	5.6 b	5.9 b	4.6 b	5.2 b	5.6 b	5.5 b	5.5 b	5.0 b	5.6 c
F3	5.9 b	6.9 a	6.4 ab	6.4 ab	4.1 b	5.3 b	5.5 b	5.5 b	6.0 ab	4.5 b	5.4 c
F4	6.9 a	7.0 a	6.0 ab	6.3 ab	6.3 a	6.5 a	6.1 ab	5.9 ab	6.7 a	7.0 a	7.0 a
F5	6.0 b	6.1 b	5.6 b	6.0 b	5.7 ab	5.9 ab	5.9 ab	5.4 b	6.2 ab	6.3 ab	6.2 bc

^†^, **, ***, significant at *p* < 0.01, and 0.001, respectively. ^‡^ Values (mean of 100 consumers) followed by the same letter, within the same column, were not significantly different (*p* < 0.05), according to LSD least significant difference test. ^¥^ (*o*) = odor; (*f*) = flavor.

**Table 3:** Results of the Cochran’s Q test for each note used in the CATA questionnaire.

Note	*p*-Values ^†^	F1	F2	F3	F4	F5
ODOR						
Fruity	NS	0.23	0.18	0.28	0.23	0.10
Vanilla	NS	0.38 a	0.32	0.40	0.35	0.30
Coffee	NS	0.42	0.35	0.32	0.35	0.55
Floral	***	0.12 ab	0.05 a	0.15 ab	0.05 a	0.30 b
Caramel	***	0.77 b	0.75 b	0.73 b	0.67 ab	0.40 a
Nutty	*	0.40 a	0.52 a	0.53 a	0.63 a	0.35 a
Mediterranean forest	NS	0.08	0.10	0.18	0.08	0.10
Raising	NS	0.45	0.52	0.58	0.62	0.60
Toffee	NS	0.57	0.38	0.43	0.42	0.35
**FLAVOR**						
Fruity	NS	0.22	0.10	0.15	0.15	0.05
Vanilla	NS	0.25	0.17	0.28	0.27	0.30
Coffee	*	0.50 ab	0.33 a	0.57 ab	0.72 b	0.55 ab
Floral	NS	0.13	0.05	0.10	0.10	0.10
Caramel	*	0.80 ab	0.67 ab	0.83 b	0.62 ab	0.55 a
Nutty	*	0.58 b	0.58 b	0.53 b	0.70 a	0.45 b
Mediterranean forest	*	0.13 ab	0.18 b	0.17 b	0.03 ab	0.00 a
Raising	NS	0.53	0.57	0.52	0.53	0.55
Toffee	NS	0.48	0.33	0.40	0.45	0.40

^†^ NS = not significant at *p* < 0.05; *, ***, significant at *p* < 0.05 and 0.001, respectively. Values followed by the same letter, within the same row, were not significantly different (*p* < 0.05).

## Data Availability

Not applicable.

## Supplementary Materials

The following are available online at https://www.mdpi.com/article/10.3390/foods10112651/s1, Figure S1: Penalty analysis of intensities of attributes different *Fondillón* wines by Spanish consumers. (Sample code effect indicated on the graphic title of each figure; “too low intensity” is indicated by the symbol “−” and “too high intensity” by the symbol “+”; Table S1: Questionnaire used in this study to establish the consumer profile.
