# Supplementary material for: Consumer Profile and Drivers Influencing Consumer Behavior towards Fondillón, a European Protected Naturally Sweet Red Wine
Source: Foods. 2021 Nov 1;10(11):2651. doi: 10.3390/foods10112651 (PMC8618339; doi:10.3390/foods10112651)
Supplement: Supplementary file 1 [file foods-10-02651-s001.zip › foods-1373463-supplementary.pdf]

Table S1. Questionnaire used in this study to establish the consumer profile.

Code	Question	
Q1	Do you know *Fondillón* wine?	
	Yes	No
Q2	Are you a *Fondillón* consumer?	
	Yes	No
Q3	If your previous answer was NO, why you do not consume it?	
	It is very expensive	It is not in the stores where I usually buy wine
	I do not like its taste	It is a wine only for special occasions
	It is hard to find	It has an excessive alcohol content
	I do not like its color	Others (please, specify):
Q4	If your previous answer was YES, how often do you consume *Fondillón*?	
	Daily	2-3 times a month
	2-3 times a week	Once a month
	Once a week	2-3 times a year
	Only in special occasions	
Q5	I drink *Fondillón* because (choose all the answers you consider appropriate):	
	It is from Alicante	It is the wine of the Count of Monte Cristo
	My grandmother already drank it	A friend recommended it to me
	It is a luxury wine	It is a local and traditional product
	I discover it while visiting a wine fair	I tried once, and I like it
Q6	What *Fondillón* brand do you know?	
	Fondillón Laudum	*Fondillón MGW*
	Fondillón Primitivo Quiles	*Fondillón Algueña*
	Fondillón Tesoro de Villena	*Fondillón Mañan*
	Fondillón Culebrón	Others (please, specify):
Q7	Which or which of them have you consumed or consume?	
	Fondillón Laudum	*Fondillón MGW*
	Fondillón Primitivo Quiles	*Fondillón Algueña*
	Fondillón Tesoro de Villena	*Fondillón Mañan*
	Fondillón Culebrón	Others (please, specify):
Q8	Where do you usually buy *Fondillón*?	
	Gourmet shop	Specialized wine shop
	Hypermarket	Straight from the winery
	Supermarket	Neighborhood store
Q9	Where do you usually consume *Fondillón*?	
	Home	Restaurant
Q10	Demographics	
Q10.1	Which is your gender?	
Q10.2	Which is your age?	
Q10.3	Which is the highest education level you have completed?	
Q10.4	How many adults live in your household including yourself?	
Q10.5	How much is your approximate annual income?	

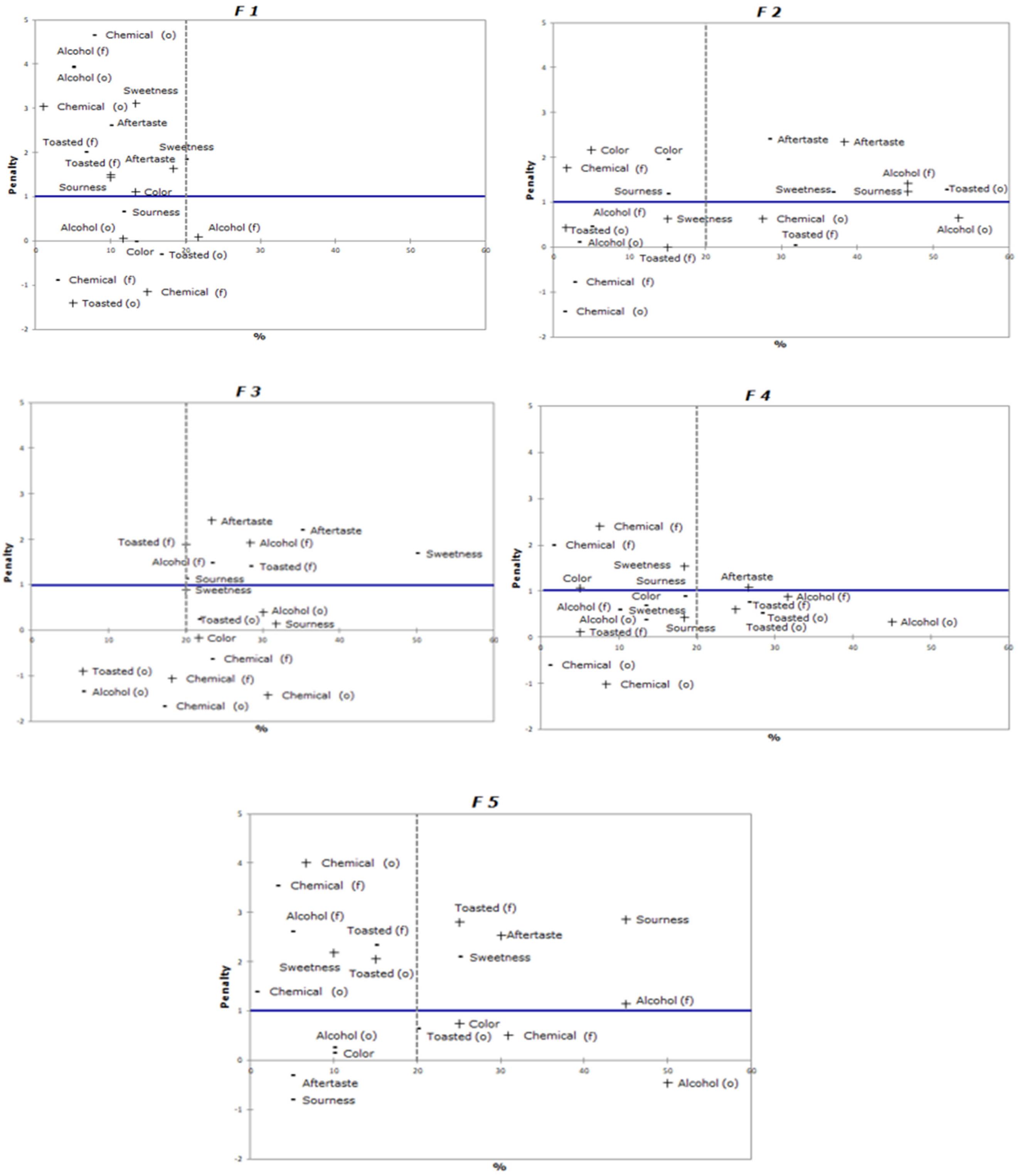

Figure S1. Penalty analysis of intensities of attributes different *Fondillón* wines by Spanish consumers. (Sample code effect indicated on the graphic title of each figure; "too low intensity" is indicated by the symbol "-" and "too high intensity" by the symbol "+"**.**
